# Mediæval Remedies for the Plague.—II

**Published:** 1898-06-25

**Authors:** 


### Modern Sociology.

### MEDIÆVAL REMEDIES FOR THE PLAGUE.—II.

The use of arsenical cakes worn under the armpits was borrowed from the practice of Paracelsus, and their efficacy was a matter of deadly debate among physicians during the plague of 1603. Their use was defended by Dr. Peter Turner, of the College of Physicians, in a little tract concerning amulets or plague cakes (1603), and seems to have found an advocate in Dr. Clarke. It was very vehemently controverted by Francis Herring, a doctor of some repute, the same year in a "Caveat given to Wearers of Empoisoned Amulets as Preservatives from the Plague." This he reinforced the following year in "A Modest Defence of the Caveat."

It appears from his tracts that plague cakes were compounded of "the powder of a toad, and of arsenic and sublimate," and that the arsenic penetrated to the system by being worn next the skin was evident by the sores frequently produced. Another writer testifies to the "foule holes made in the breast of those that have worn them." The value of arsenic as a preventive medicine in times of infection is now generally recognised, but it was defended in those days by strange arguments, and undoubtedly owed much of its popularity to the element of mystery and magic in which the amulet was wrapped. The most scientific were driven to plead that arsenic profited by reason of the spirits of gold which it contained, and to this Herring retorted that he had consulted a cunning work-master in metals in London who affirmed that "there was so much gold in arsenic as in a rat." There is reason to suppose that the wearing of an amulet believed to confer immunity from the disease may have been of service to many, independently of its medicinal properties, in a season when panic was one of the deadliest dangers to be shunned.

The plague waters, which form a common feature in all books on medicine (and even on cookery) down to the middle of the eighteenth century, were nearly all designed for prevention rather than cure. They are mere herbal concoctions, for the most part of a very innocent nature, and were resorted to on the slightest rumour of infection long after the plague had ceased to recur in England. Concerning the actual treatment of the disease after it had once seized on the patient very little information is to be gained. By far the greater number of persons perished without any species of relief being attempted for them. Many instances are on record of plague victims in districts yet uninfected being turned out to die in the open, deprived of the commonest offices of charity. In the worst seasons of epidemic hundreds perished in their own homes deserted by all. At the pest-houses some course of treatment must have been attempted, but the only record preserved with regard to them is the weekly tale of deaths, and that can tell little, since the amount of accommodation is not known. In 1603 three hospitals were available for plague cases, the pest-houses in Finsbury Fields and Tothill Fields, and the Bridewell, near Fleet Street, but it does not appear that they were ever resorted to of free will. Domestic servants, apprentices, and dependents were often compulsorily removed to them by masters who dreaded infection, and they formed at least a shelter for the homeless poor to die in.

It has been seen that the doctors, as a rule, preferred to follow their richer patients out of town and practice with preventive drugs. In cases where, at a great price to persons of consideration, the physician lent his presence to the infected chamber, it was his custom to stand near the door sniffing essences and prescribe acccrding to a thoroughly conventional routine which varied little from one century to another. As a matter of fact, when the patient was actually attacked, all was practically over in the worst seasons, and when the epidemic was subsiding nature worked her own cure unless the constitution was undermined by mistaken treatment. The usual plan was to begin with "letting blood," and this operation, performed by the apprentice or assistant, without whom the physician of repute never stirred, entailed no actual contact with the patient upon the great man. Herbal waters brewed of endive, century, ivy-berries, &c., were next freely administered to induce a heavy sweat, and a favourite drug at the commencement of the attack was saffron enclosed in an egg-shell, roasted and brazed in a mortar and drunk with ale. Cedar wood, juniper, rosemary, and other sweet-smelling things were ordered to be burnt in the sick-room, and sometimes under the bed, that the patient might be disinfected; and one writer, in 1594, directs that a basin of new milk be left standing in the room, which after two days will draw all the infection to it. Three or four large onions laid on the ground will effect the same purpose, but they must be let lie there for ten days and then buried deep in the ground.

These measures having been taken, the next step was directed to the treatment of the plague bubo, and here authorities differed widely; some urged its immediate incision because "if nature be weak and not able to expel the venom fast enough, by insensible transpiration the venom returneth back to the heart, and so presently destroyeth nature." Others, again, believed the use of the lancet to be fatal, and relied on poultices made of onions or bread, which were to be buried after use, partly, no doubt, as a wise method of precaution, but partly also with reference to that leaning towards magic noticeable in so much of the medical practice of the period. A barbarous species of poultice much in vogue was to "set hennes on the sore—they will draw out the venom and die." The speedy death of the birds, whose place was supplied by others, was used as an argument in favour of the contagiousness of the plague, a point then, as now, a matter of doubt to some of those whose observations had been most close.

A curious prescription, which it may be believed only the "richer sort" could compass in times of panic, was for a quilt "to be laid on the heart of the sick after sweating." It was compounded of flowers of water-lilies, borage, buglosse, red rose leaves, balm, rosemary, aloes, cloves, juniper seeds, saffrons, bone of deer's heart, &c., "quilted in crimson or scarlet coloured taffeta." Such were the methods and resources of the regular physician.

